# Supplementary material for: Increased Respiratory Drive after Prolonged Isoflurane Sedation: A Retrospective Cohort Study
Source: J Clin Med. 2022 Sep 15;11(18):5422. doi: 10.3390/jcm11185422 (PMC9504554; doi:10.3390/jcm11185422)
Supplement: Supplementary file 1 [file jcm-11-05422-s001.zip › jcm-1864520-supplementary.pdf]

Analgesia, Sedation and Delirium Therapy

Version	**1**
Distributor	
Scope	**Intensivmedizin**
This AA applies from	[Release Date]
This AA replaces the version of	[QM release date previous version]
Enclosure	

	Name, function, date	Signature
Created	**Conrad, David - ; 03.09.2019**	
Checked	**Meiser, Andreas Dr. med. - - 16.09.2019**	
Approved (internal)	[QM approved internally by]	
Approved (External)	[QM approved externally by]	
Released	**Schröder, Matthias Dr. med. - - 24.09.2019**	

Content

1 General 3
2 Goal/Purpose 3
3 Scope 3
4 Responsibilities 3
5 Principles 3
6 Analgesia 4
7 Sedation 5
8 Delirium 6
8.1 Prevention of delirium: 6
8.2 Principles for the therapy of delirium: 7
8.3 Drug therapy proposal 7
9 Attachments 8
9.1 Richmond Agitation and Sedation Scale: 8
9.2 Differential diagnosis of the organic causes of delirium: 8
10 Applicable documents 8
11 Modification history 8
12 List of abbreviations 9

1 General

"The intensive care patient should be awake, alert, and without pain, anxiety and delirium in order to be able to participate actively in his treatment and recovery." The S3 guideline "Analgesia, Sedation and Delirium Management in Intensive Care Medicine" serves as the basis for this Standard Operating Procedure (SOP).

2 Goal/Purpose

The goals of this SOP 'Analgesia, Sedation and Delirium Therapy' are adequate protocol-based analgesia and sedation of the intensive care patient. Likewise, the prevention, as well as the consistent treatment of delirium in patients of the IOI is an important goal. For this purpose, patient-specific therapy goals are defined (e.g. in interprofessional rounds), prescribed by the physician and controlled and documented by the nurse.

3 Scope

This SOP applies to the entire IOI.

4 Responsibilities

physicians: Selection of medications, therapy indication and definition of therapeutic goals Caregivers: Achieving therapeutic goals, documentation of drug dosages and effects (pain, sedation, delirium scores)

5 Principles

- Regular monitoring of pain, depth of sedation and delirium
- Muscle relaxants should be avoided whenever possible and should not be used regularly outside the induction of anesthesia.
- Syringes with a mixture of drugs shall not be used.
- The effect and side effect of procedures and medications used must be known to the responsible physicians.
- As few medications as possible should be used. In this way, unwanted interactions can be avoided.

6 Analgesia

- Adequate analgesia is an important cornerstone in the prevention of delirium and treatment/rehabilitation of intensive care patients. The perception of pain varies greatly from patient to patient.
- A validated pain scale (NRS-V, BPS) determines the pain level at least every 8 hours. Objective Numerical Rating Scale (NRS) <4
- The principles of the WHO step-by-step scheme apply.
- The opioid of choice is hydromorphone. It can be used as a PCA/NCA with and without continuous infusion rate and as an oral medication.
- The opioid of choice for the intubated patient is remifentanil. The continuous infusion via a syringe pump is carried out according to the hospital standard.
- A PCA with continuous infusion rate is not permitted on the normal ward. Before transfer, pain therapy must therefore be adjusted (by means of oral sustained-release drug preparations) in such a way that a continuous infusion is no longer required.
- The NSAIDs of choice are metamizole and ibuprofen.
- Regional anesthesia – close to the spinal cord, as well as peripheral – should be used if available. After proper informed consent and indication, it is generally possible to perform regional anesthesia procedures in the ICU. With regard to the system and the dosage of medication, departmental standards apply.
- Before nursing care, medical or physiotherapeutic measures, bolus administrations (of the PCA/NCA or the regional anesthesia procedure) are useful. An increase in the infusion rate is not reasonable. However, bolus administration of remifentanil should not be carried out.
- PCA syringe changes must be documented in the electronic patient chart. Documentation of narcotic drugs is obligatory.
- Simply prescribing PCA pumps – especially with continuous infusion rates – does not make sense. Acute (postoperative) pain should be treated by targeted administration of intravenous hydromorphone at the bedside.
- A differentiated pain therapy for special forms of pain may be necessary, but this is not within the scope of this SOP.

7 Sedation

- The need for sedation, the depth of sedation and the type of sedation shall be determined individually for each patient.
- In general, patients should only be sedated as deeply as necessary. Unnecessarily long or too deep sedation has a negative impact on outcome.
- Exceptions are, of course, severe life-threatening conditions such as traumatic brain injuries with increased intracranial pressure or continuous lateral rotational therapy in specially designed beds (Rotorest®).
- The sedation depth is monitored at least every 8 hours with the RASS score.
- Target for intubated patients: -3 to 0; for non-intubated: -1 to 0 (during daytime)
- The mere presence of an endotracheal tube is not an indication for deep sedation.
- Standard sedation for invasively ventilated patients is the combination of remifentanil plus propofol or remifentanil plus isoflurane.
- Propofol should not be used for more than 7 days and at concentrations of more than 4mg per kg body weight per hour.
- Isoflurane is preferable to propofol in patients with pulmonary dysfunction, high sedative needs and/or in patients with difficult-to-achieve spontaneous breathing.
- Sedation windows, if no contraindication, should be performed once every 24 hours. For neurological assessment in case of deep sedation (e.g. polytrauma or resuscitation), a sedation window after 24 hours is mandatory!
- Intubated patients should breathe spontaneously with adequate support by the ventilator in (CPAP+ASB)
- For non-intubated or tracheotomized patients, sedation with propofol is usually not necessary. Analgesia is usually sufficient. If sedation is indicated, the medication depends on the cause, see below.
- If sedation is indicated, the dose and depth of sedation should be adjusted to the day/night rhythm.
- In certain patients (e.g. substance abuse), further drugs such as midazolam or ketamine can be considered both as a bolus and as a continuous administration via syringe pump in order to achieve the desired depth of sedation.
- When deep sedation is performed, processed EEG systems are recommended.

8 Delirium

- Delirium is very common in intensive care patients.
- Delirium increases morbidity (including more ventilation days, longer length of stay, limited cognitive performance even after discharge) as well as mortality (up to 3-10 times)! Thus, prevention of delirium plays a particularly important role!

8.1 Prevention of delirium:

- In order to detect delirium at an early stage, delirium screening is carried out every 8 hours using the Intensive Care Delirium Screening Checklist (ICDSC).
- Early mobilization and the consistent adjustment of the day-night rhythm are essential. Suitable mobilization goals are set for each patient. From passive movement to mobilization with the patient standing or walking, a number of suitable patient-centered measures are possible. Use of the mobilization chair Thekla® is recommended, as this is possible at an early stage and allows patients to re-orientate and participate in daily-living activities.
- Hearing and visual aids must be handed over to the patient as soon as reasonable.
- Whenever possible, patients should be positioned in a way that allows them to overlook their environment and to see, what is going on. Social deprivation should be avoided.
- Cognitive stimulation: Patients should be able to watch the time. Time and date, as well as any planned measures should be communicated to the patients
- At night, sufficient darkness and silence should be ensured.
- Suitable and sufficient analgesia must be guaranteed.
- If sedation is required, this should only be as deep as necessary.
- In order to differentiate hypoactive delirium from a nonconvulsive epileptic state, an EEG examination may be useful.
  (This is requested via the neurosurgical department, interpretation is done by neurologists)

8.2 Principles for the therapy of delirium:

- There is no curative pharmacological therapy of delirium. This makes prevention all the more important.
- Any organic causes must be ruled out and treated (see mnemonic below).
- The therapy should be based on individual symptoms and suspected causes.
- Generally, all preventive measures mentioned above are also an important part of non-pharmacological therapy.

8.3 Drug therapy proposal

Agitation and pronounced vegetative symptoms	**alpha-2 agonists**: clonidine as a bolus; or continuous infusion.<br>Continuous infusion of dexmedetomidin: 0,2-1,4µg/kg/min.<br>Caveat: Bradycardia.<br>In case of severe acute agitation, it may be necessary to use short-acting sedative drugs, e.g. midazolam or propofol. Any continuous infusion of benzodiazepines (or of propofol in non-intubated patients) must be consulted with a medical specialist (Facharzt)!
(Alcohol) Withdrawal delirium	alpha-2 agonists as continuous infusion, if necessary combined with long-acting benzodiazepines
Productive psychotic symptoms	**Acute:** intravenous Haloperidol, not exceeding 15mg/day (<10 mg/d in geriatric patients!);<br>Oral Melperone 20-50mg (available as syrup)<br>**Longer term:** oral risperidone, olanzapine, or quetiapine
Central anticholinergic syndrome	Physostigmine
Anxiety	Benzodiazepines for acute panic attacks,<br>antidepressants,<br>but above all personal attention.

9 Attachments

9.1 Richmond Agitation and Sedation Scale:

Physical stimulation is done by touching the forearm, shaking, star rubbing. A pain stimulus is not required!

+ 4	Very belligerent
+ 3	Very agitated
+ 2	Agitated
+ 1	Restless
0	Awake and quiet
- 1	sleepy, eye contact > 10s after speech
- 2	Light sedation, eye contact < 10s after speech
- 3	Moderate sedation, response without eye contact after speech
- 4	Deep sedation, response to physical stimulation
- 5	Not arousable, no response to physical stimulation

The patient is addressed with: The patient's name, "Please open your eyes", "Please look at me"

9.2 Differential diagnosis of the organic causes of delirium:

I	Infection
W	Withdrawal
A	Acute Metabolic
T	Trauma
C	CNS-pathology
H	Hypoxia
D	Deficiencies
E	Endocrinopathies
A	Acute Vascular
T	Toxins/Drugs
H	Heavy Metals

10 Applicable documents

SOP of the QM Portal for the IOI

11 Modification history

[Enter text here]

12 List of abbreviations

SOP	Standard Operating Procedure
RASS	Richmond Agitation and Sedation Scale
IOI	Interdisciplinary Operative Intensive Care Unit
NRS	Numeric Rating Scale
BPS	Behavioral Pain Scale
PCA	patient controlled analgesia
NCA	nurse controlled analgesia
NSAID	nonsteroidal anti-inflammatory drugs
ICU	intensive care unit
WHO	World Health Organization
CPAP	continuous positive airway pressure
ASB	assisted spontaneous breathing (=pressure support)
ICDSC	Intensive Care Delirium Screening Checklist
EEG	electroencephalography
QM	Quality Management
